# Primary cutaneous diffuse large B‐cell lymphoma, leg type with *MYC/BCL2/BCL6* overexpression

**DOI:** 10.1002/jha2.364

**Published:** 2021-12-18

**Authors:** Yosuke Okada, Takehiro Sone, Kohei Takada, Hiraku Ogata, Keita Saito, Fumihiko Kimura

**Affiliations:** ^1^ Division of Haematology Department of Internal Medicine National Defense Medical College Hospital Saitama Japan

A 58‐year‐old man came to our hospital with a 2‐month history of progressive cutaneous tumor exuding foul smell. The lesions were mainly located in the lower legs (Figure 1A,B), but directly infiltrated into muscles and bones, as shown by computed tomography (CT) scan. Tumor biopsy demonstrated diffuse proliferation of large cells. Immunohistochemistry analysis showed positive expression of CD20, CD79a, and monotypic immunoglobulin λ light‐chain genes with overexpression of *MYC*, *BCL2*, and *BCL6*. We diagnosed him with primary cutaneous diffuse large B‐cell lymphoma (PC‐DLBCL), leg type. In addition, he was histologically classified as triple‐expressor lymphoma.

He was treated with one cycle of cyclophosphamide, doxorubicin, vincristine, and dexamethasone (CHOP) followed by six cycles of dose‐adjusted etoposide, prednisone, vincristine, cyclophosphamide, doxorubicin, and rituximab (DA‐EPOCH‐R). The lesions appeared to be eliminated, but positron emission tomography/CT showed residual disease in lower left leg. Salvage chemotherapy met with poor response, and unfortunately, he died 19 months after the initial diagnosis.

**FIGURE 1:**
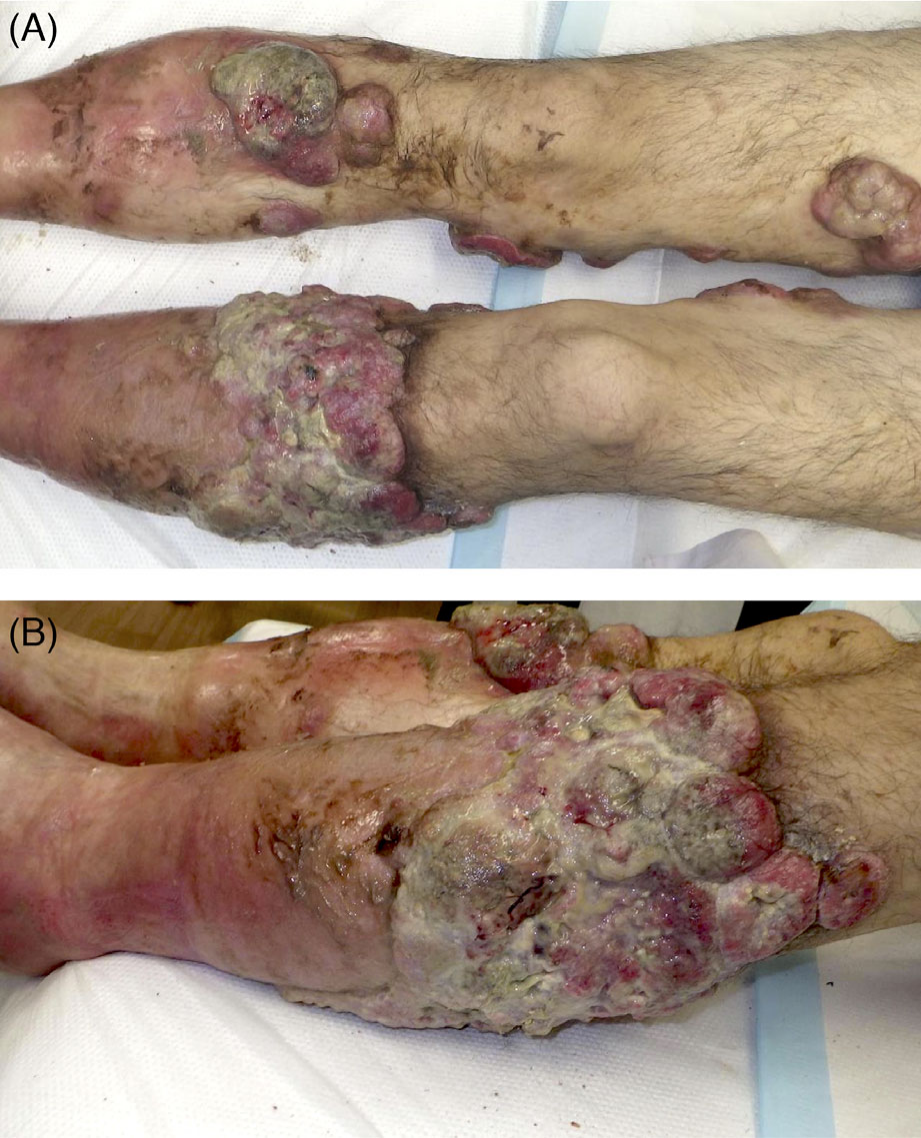
PC‐DLBCL in the lower legs

## AUTHOR CONTRIBUTIONS

Yosuke Okada treated the patient and wrote the paper. Takehiro Sone, Kohei Takada, Hiraku Ogata, and Keita Saito treated the patient. Fumihiko Kimura treated the patient and advised on the paper.

## CONFLICT OF INTEREST

The authors declare no conflict of interest.

